# Is remnant pancreatic cancer after pancreatic resection more frequent in early‐stage pancreatic cancer than in advanced‐stage cancer?

**DOI:** 10.1002/ags3.12340

**Published:** 2020-05-05

**Authors:** Yoshihiro Miyasaka, Takao Ohtsuka, Ryuichiro Kimura, Ryota Matsuda, Yasuhisa Mori, Kohei Nakata, Masato Watanabe, Yoshinao Oda, Masafumi Nakamura

**Affiliations:** ^1^ Department of Surgery and Oncology Graduate School of Medical Sciences Kyushu University Fukuoka Japan; ^2^ Department of Surgery Fukuoka University Chikushi Hospital Chikushino Japan; ^3^ Department of Anatomic Pathology Graduate School of Medical Sciences Kyushu University Fukuoka Japan

**Keywords:** neoplasm, neoplasm staging, pancreatectomy, pancreatic cancer, recurrence, second primary

## Abstract

**Aim:**

As the prognosis of patients who undergo resection for pancreatic cancer has improved, reports of remnant pancreatic cancer after pancreatic cancer resection have been increasing. Previous studies regarding early‐stage pancreatic cancer showed a high incidence of remnant pancreatic cancer in these patients. The aim of this study was to investigate the incidence of remnant pancreatic cancer according to the degree of progression of the initial pancreatic cancer.

**Methods:**

Patients who underwent partial pancreatic resection for primary pancreatic cancer were retrospectively reviewed and divided into an early‐stage group and an advanced‐stage group according to the stage of the initial cancer. Patient characteristics and long‐term outcomes, including development of remnant pancreatic cancer, were compared between the two groups.

**Results:**

This study included 321 patients who underwent partial pancreatectomy for pancreatic cancer; 32 patients in the early‐stage group and 289 patients in the advanced‐stage group. Remnant pancreatic cancer developed in 19 patients (5.9%); seven patients (21.9%) in the early‐stage group and 12 patients (4.5%) in the advanced‐stage group. The cumulative incidence of remnant pancreatic cancer according to the Kaplan–Meier method was comparable between the two groups (5‐year cumulative incidence: 20.6% vs 9.9%, early‐stage group vs advanced‐stage group; *P* = .1827).

**Conclusion:**

Our results suggested that the potential for developing remnant pancreatic cancer was comparable between the early‐stage and the advanced‐stage groups. Therefore, the incidence of remnant pancreatic cancer may increase along with improved pancreatic cancer treatment.

## Graphical abstract

Remnant pancreatic cancer occasionally develops after pancreatic resection for pancreatic cancer. Although the proportion of patients who develop remnant pancreatic cancer was larger in early‐stage pancreatic cancer than advanced‐stage cancer, the potential for developing remnant pancreatic cancer was comparable between early‐stage cancer and advanced‐stage cancer.

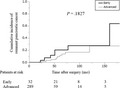

## 1 INTRODUCTION

Pancreatic cancer is the most lethal malignancy of the digestive system, with a 5‐year relative survival rate of only 9%.^1^ Currently, the only way to cure pancreatic cancer is complete surgical resection of a primary lesion, if the lesion is resectable. Because of early detection and improved multidisciplinary treatments, the prognosis of patients who undergo resection for pancreatic cancer has improved.^2^, ^3^ While the number of long‐term survivors after surgery for pancreatic cancer has been increasing, reports of patients who develop metachronous cancer in the remnant pancreas have also increased.^4^ The proportion of patients who develop remnant pancreatic cancer after resection of pancreatic cancer has been reported to range from 0.7% to 26.7%.^4^ Notably, some studies focusing on early‐stage pancreatic cancer (stage 0‐I) patients showed a high incidence of remnant pancreatic cancer.^5^, ^6^ However, no study has compared the incidence of remnant pancreatic cancer after resection of early‐stage pancreatic cancer with that of advanced‐stage pancreatic cancer.

In this study, to investigate the incidence of remnant pancreatic cancer according to the degree of progression of the initial pancreatic cancer, we compared the long‐term outcomes of patients who underwent partial pancreatic resection for early‐stage pancreatic cancer to outcomes of patients who had surgery for advanced‐stage pancreatic cancer.

## 2 METHODS

### 2.1 Study design

This study was approved by the Ethics Committee of Kyushu University and conducted according to the Helsinki Declaration. Patients who underwent pancreatic resection for primary pancreatic cancer at the Department of Surgery and Oncology, Kyushu University, between 1992 and 2013 were retrospectively reviewed. Patients who underwent neoadjuvant therapy for pancreatic cancer and those who underwent total pancreatectomy were excluded. This study was focused on conventional pancreatic ductal adenocarcinoma (PDAC), PDAC derived from intraductal papillary mucinous neoplasm (IPMN), PDAC derived from intraductal tubulopapillary neoplasm, and PDAC derived from mucinous cystic neoplasm; acinar cell carcinomas were excluded. Cases of PDAC concomitant with IPMN were included in the study. Clinicopathological data including age, sex, tumor location, existence of concomitant IPMN, date of surgery, surgical procedures, pathological outcomes (histopathological diagnosis, resection margin status, TNM status according to the 7th edition of the Union for International Cancer Control classification^7^), adjuvant therapy, development of extrapancreatic recurrence and remnant pancreatic cancer, survival, and date of last follow‐up were collected from medical records, operation records, and pathological reports. Follow‐up data until December 2018 were collected. Remnant pancreatic cancer was diagnosed according to the following criteria, based on previous reports^8^, ^9^: (a) the tumor developed within the remnant pancreas; (b) pathologically negative pancreatic cut margin at the initial pancreatic resection; (c) more than a 12‐month interval between the initial pancreatic resection and the secondary lesion; and (d) pathological or cytological diagnosis of carcinoma or radiological findings strongly suggesting malignancy, such as invasion to the surrounding tissue or distant metastasis. Patients with initial pancreatic cancer at stage 0, IA, and IB were designated as the early‐stage group, while patients with stage IIA‐IV pancreatic cancer were designated as the advanced‐stage group. The patient characteristics and long‐term outcomes of the early‐stage group were analyzed and compared with those of the advanced‐stage group.

### 2.2 Statistical analyses

All statistical analyses were performed using JMP statistical software version 14.0 (SAS Institute). Continuous variables were compared by the Student's t‐test or the Mann–Whitney U‐test. Categorical variables were compared by the chi‐square test or Fischer's exact test. The disease‐free interval was defined as the duration between the date of the initial operation and the date of diagnosis of extrapancreatic recurrence or remnant pancreatic cancer. Overall survival (OS) was defined as the duration between the date of initial surgery and the date of death or last follow‐up. The Kaplan–Meier method was used to estimate OS and the cumulative incidence of extrapancreatic recurrence or remnant pancreatic cancer, and the log‐rank test was used for comparisons. A two‐sided *P*‐value of <.05 was considered statistically significant.

## 3 RESULTS

**FIGURE 1:**
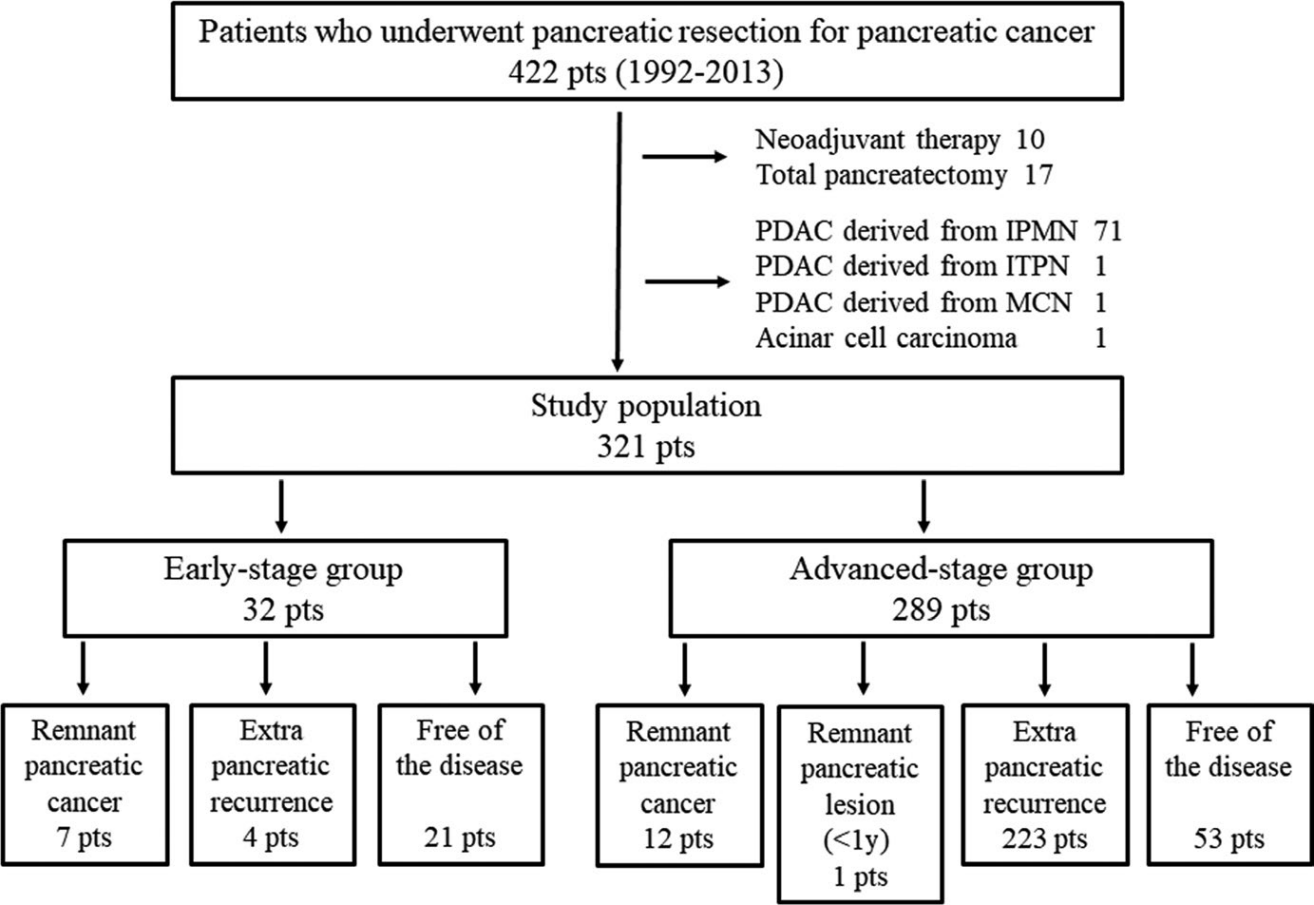
Flow diagram of the study. IPMN, intraductal papillary mucinous neoplasm; ITPN, intraductal tubulopapillary neoplasm; MCN, mucinous cystic neoplasm; PDAC, pancreatic ductal adenocarcinoma

**FIGURE 2:**
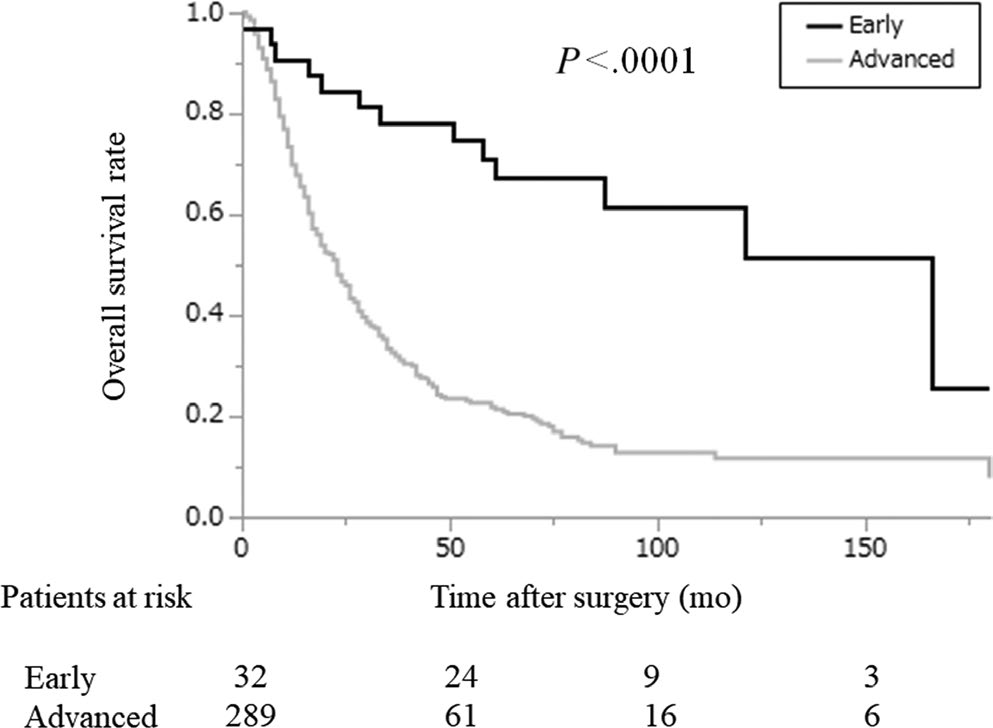
Overall survival rates of pancreatic cancer patients after initial pancreatic resection for pancreatic cancer according to the degree of progression. Black line: early‐stage group; gray line: advanced‐stage group

Among the 422 patients who underwent pancreatic resection for pancreatic cancer, 321 were included in this study (Figure 1). Among the 321 patients, 32 were classified as the early‐stage group, and 289 patients were classified as the advanced‐stage group. The characteristics of the patients in the two groups are shown in Table 1. Age and the male‐female ratio were comparable between the two groups. Cancer in the pancreatic body‐tail was more frequent in the early‐stage group. Concomitant IPMN was also recognized more frequently in the early‐stage group. The observation period was significantly longer in the early‐stage group than in the advanced‐stage group (70.5 months vs 21 months, early‐stage group vs advanced‐stage group; *P* < .0001). OS was also significantly longer in the early‐stage group than in the advanced‐stage group (median survival time, 166 months vs 23 months, early‐stage group vs advanced‐stage group; *P* < .0001; Figure 2).

**TABLE 1:** Characteristics of patients who underwent partial pancreatectomy for pancreatic cancer

	Early‐stage group (n = 32)	Advanced‐stage group (n = 289)	*P* value
Age (years; median)	68 (43‐82)	67 (36‐88)	.7878
Sex (male/female)	18/14	180/109	.5082
Location (head/body‐tail)	13/19	195/94	.0034
Procedure (PD/DP)	13/19	196/93	.0032
Concomitant IPMN (yes/no)	9/23	22/267	.0014
Stage (0/IA/IB/IIA/IIB/III/IV)	9/17/6/0/0/0/0	0/0/0/61/215/4/9	<.0001
Margin status (R0/R1/R2)	32/0/0	207/77/5	<.0001
Adjuvant therapy (yes/no)	12/20	233/56	.0231
Observation period (months; median)	70.5 (0‐197)	21 (1‐222)	<.0001

Abbreviations: DP, distal pancreatectomy; IPMN, intraductal papillary mucinous neoplasm; PD, pancreatoduodenectomy.

**FIGURE 3:**
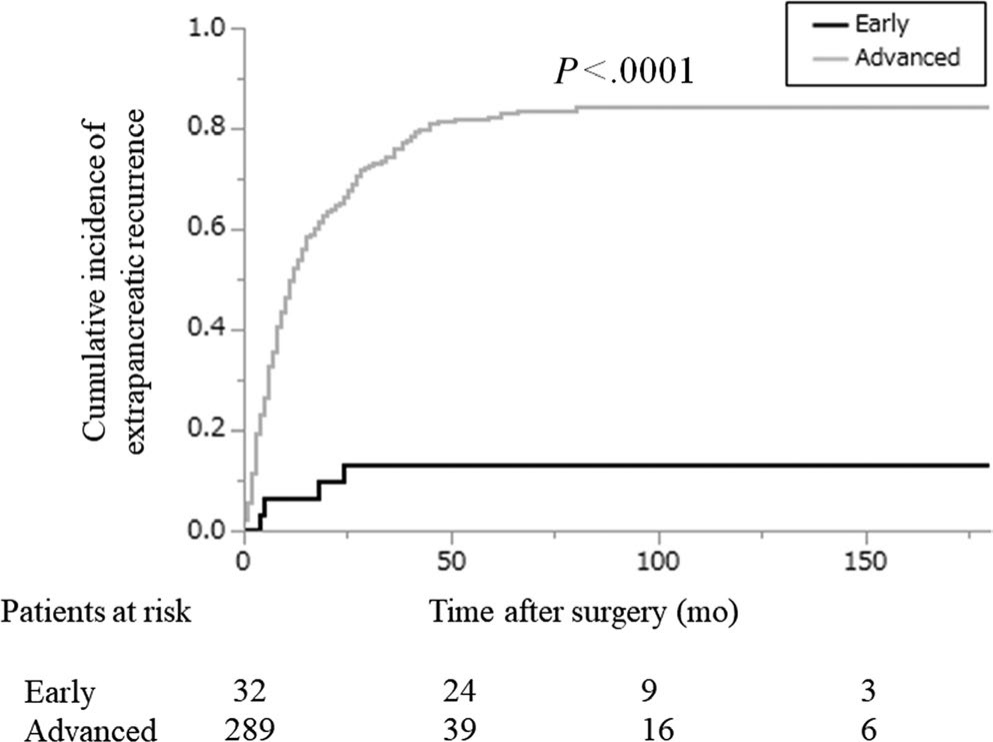
Cumulative incidence of extrapancreatic recurrence after initial pancreatic resection for pancreatic cancer according to degree of progression. Black line: early‐stage group; gray line: advanced‐stage group

Extrapancreatic recurrence was observed in 227 patients (70.7%): four patients in the early‐stage group and 223 patients in the advanced‐stage group. Compared with the early‐stage group, the advanced‐stage group showed a higher cumulative incidence of extrapancreatic recurrence (5‐year cumulative incidence: 13.3% vs 82.3%, early‐stage group vs advanced‐stage group; *P* < .0001; Figure 3).

**FIGURE 4:**
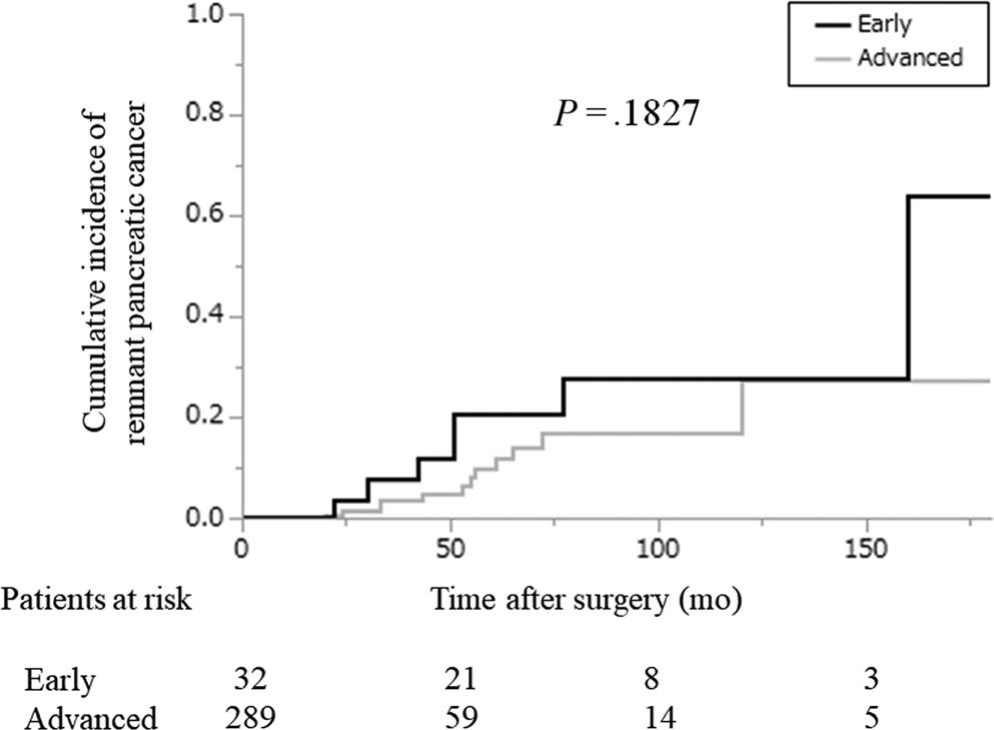
Cumulative incidence of remnant pancreatic cancer after initial resection for pancreatic cancer according to the degree of progression. Black line: early‐stage group; gray line: advanced‐stage group

**FIGURE 5:**
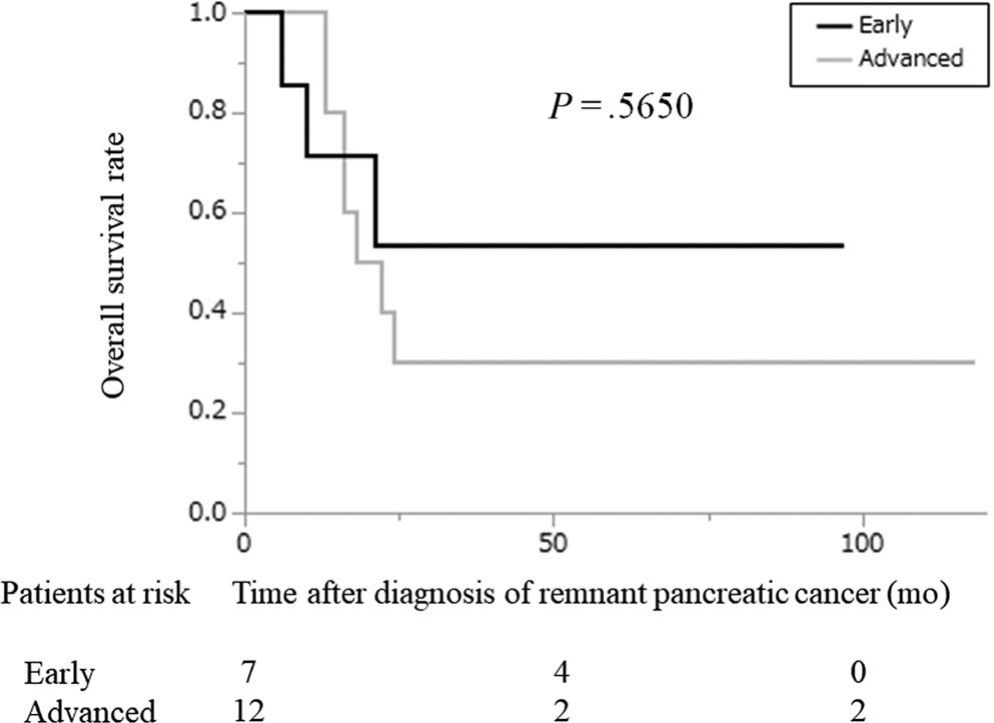
Overall survival of patients with remnant pancreatic cancer after a diagnosis of remnant pancreatic cancer according to the degree of progression. Black line: early‐stage group; gray line: advanced‐stage group

**FIGURE 6:**
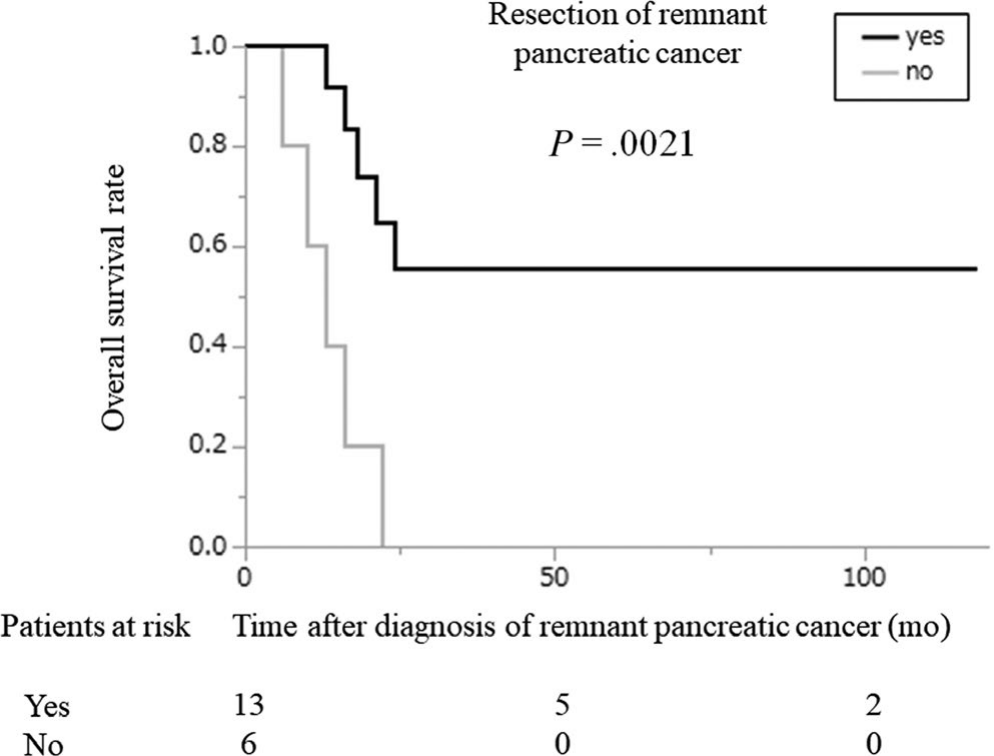
Overall survival of patients with remnant pancreatic cancer after a diagnosis of remnant pancreatic cancer according to resection for remnant pancreatic cancer. Black line: patients who underwent resection for remnant pancreatic cancer; gray line: patients who did not undergo resection for remnant pancreatic cancer

Remnant pancreatic cancer developed in 19 patients (5.9%): seven patients in the early‐stage group and 12 patients in the advanced‐stage group (Figure 1). We did not include one patient who developed a remnant pancreatic lesion that was pathologically diagnosed as adenocarcinoma that appeared 8 months after the initial pancreatic resection. The cumulative incidence of remnant pancreatic cancer was comparable between the two groups (5‐year cumulative incidence: 20.6% vs 9.9%, early‐stage group vs advanced‐stage group; *P* = .1827; Figure 4). The characteristics of the patients who developed remnant pancreatic cancer are shown in Table 2 and were comparable between the two groups except for stage. Although two patients with remnant pancreatic cancer in the advanced‐stage group underwent R1 resection in the initial operation, pancreatic cut margins were negative. The interval between the initial pancreatic resection and the diagnosis of remnant pancreatic cancer was also comparable between the two groups (51 months vs 54 months, early‐stage group vs advanced‐stage group; *P* = .9053). Thirteen of the 19 patients underwent total pancreatectomy for remnant pancreatic cancer. In all 13 patients, the pathological diagnosis of the remnant pancreatic cancer was conventional PDAC. Our indication for surgical resection of remnant pancreatic cancer was the same as for primary pancreatic cancer, and the reasons for non‐surgical treatment were metastatic disease in two patients, locally advanced disease in one patient, advanced age (90 years old) in one patient, and patient refusal in two cases. The resection rates of remnant pancreatic cancer were comparable between the two groups (71% vs 67%, early‐stage group vs advanced‐stage group; *P* > .9999). The prognosis after both the initial pancreatic resection and the diagnosis of remnant pancreatic cancer in the early‐stage group tended to be more favorable compared with the prognosis in the advanced‐stage group, although the difference was not significant (median survival time after initial pancreatic resection, 166 vs 81 months, early‐stage group vs advanced‐stage group; *P* = .2407 and median survival time after the diagnosis of remnant pancreatic cancer, not reached vs 20 months, early‐stage group vs advanced‐stage group; *P* = .5650; Figure 5). Survival after the diagnosis of remnant pancreatic cancer was significantly longer in patients who underwent resection for remnant pancreatic lesions than in those who did not, although survival after the initial pancreatic resection failed to show statistical significance (median survival time after the initial pancreatic resection, not reached vs 84 months, early‐stage group vs advanced‐stage group; *P* = .6767 and median survival time after the diagnosis of remnant pancreatic cancer, not reached vs 13 months, early‐stage group vs advanced‐stage group; *P* = .0021; Figure 6).

**TABLE 2:** Characteristics of patients with remnant pancreatic cancer

	Early‐stage group (n = 7)	Advanced‐stage group (n = 12)	*P* value
Age (y.o.; median)	59 (54 ‐ 69)	63 (37 ‐ 84)	.2889
Sex (Male/Female)	5/2	7/5	.6562
Location (head/body‐tail)	1/6	4/8	.6027
Procedure (PD/DP)	1/6	4/8	.6027
Concomitant IPMN (yes/no)	3/4	4/8	>.9999
Stage (0/IA/IB/IIA/IIB/III/IV)	2/4/1/0/0/0/0	0/0/0/2/10/0/0	.0008
Margin status (R0/R1/R2)	7/0/0	10/2/0	.5088
Adjuvant therapy (yes/no)	5/2	10/2	.6027
Disease‐free interval^a^ (month; median)	51 (22‐160)	54 (20‐120)	.8657
Resection of remnant pancreatic cancer (yes/no)	5/2	8/4	>.9999

Abbreviations: DP, distal pancreatectomy; IPMN, intraductal papillary mucinous neoplasm; PD, pancreatoduodenectomy.

^a^ Interval between the initial pancreatic resection and the diagnosis of remnant pancreatic cancer.

## 4 DISCUSSION

This cohort study examining patients who underwent partial pancreatic resection according to the degree of progression of the initial cancer revealed the following findings: (a) the cumulative incidence of remnant pancreatic cancer was comparable between the early‐stage group and the advanced‐stage group, and (b) surgical resection of remnant pancreatic cancer was associated with favorable long‐term outcomes.

The comparable cumulative incidence between the two groups suggested that the remnant pancreas of patients in the advanced‐stage group had the same risk of developing metachronous cancer as in the early‐stage group. It is reasonable to consider that the degree of progression of the initial pancreatic cancer does not influence cancer susceptibility in the remnant pancreas. However, the proportion of patients who developed remnant pancreatic cancer in the early‐stage group was similar to the proportions in previous studies regarding early‐stage pancreatic cancer,^5^, ^6^ and was significantly larger compared with the advanced‐stage group in our study (21.9% vs 4.2%; *P* = .0011). The significantly smaller proportion of patients who developed remnant pancreatic cancer in the advanced‐stage group is likely attributable to shorter observation periods because of the lower rate of survival in the advanced‐stage group. In other words, if the two groups had shown similar survival rates and observation periods, the proportion of patients who would have developed remnant pancreatic cancer would have been comparable between the two groups. Another possible reason for the larger proportion of patients who developed remnant pancreatic cancer in the early‐stage group is the larger proportion of patients with cancer in the pancreatic body‐tail in the early‐stage group. Generally, pancreatic cancer occurs more frequently in the pancreatic head,^3^ suggesting that the pancreatic head is more susceptible to carcinogenesis. It is suspected that remnant pancreatic cancer develops more frequently after resection of cancer in the pancreatic body‐tail. In this study, indeed, remnant pancreatic cancer developed more frequently after resection of cancer in the pancreatic body‐tail (14/19) in spite of the smaller proportion of cancers in the pancreatic body‐tail (113/321). The cumulative incidence of remnant pancreatic cancer was higher in the early‐stage group, although the difference was not significant. The difference would have been smaller if the proportions had been comparable between the two groups. Further analyses such as multi‐institutional surveillance are necessary to prove the hypothesis that remnant pancreatic cancer develops more frequently after resection of cancer in the pancreatic body‐tail.

Two potential mechanisms underlying the development of remnant pancreatic cancer after partial pancreatic resection for pancreatic cancer have been proposed. One is the recurrence of the initial pancreatic cancer, and the other is the metachronous development of a new primary cancer.^4^ Several studies that compared genetic mutations between the initial and remnant pancreatic lesions supported the presence of both patterns.^8^, ^10^, ^11^ Because of the aggressive nature of pancreatic cancer, approximately 80% of patients who undergo surgery for pancreatic cancer develop recurrence.^12^, ^13^ Although the main sites of recurrence are the pancreas bed, liver, peritoneum, and lung, recurrence is also detected in the remnant pancreas.^12^, ^13^, ^14^ Several pathways underlying how cancer cells of the initial lesion reach the remnant pancreas have been suggested, such as a positive margin, hematogenous metastasis, lymphogenous spread, peritoneal dissemination, and intraductal dissemination.^8^, ^15^ However, other reports have found that pancreatic cancer often shows multifocal lesions. Histopathological examination of pancreases retrieved in total pancreatectomy for pancreatic cancer has revealed that 20%‐32% of patients had multifocal cancer.^16^, ^17^ Multifocal lesions may develop metachronously after partial pancreatic resection for pancreatic cancer. Gotoh et al divided remnant pancreatic cancer cases into cases with recurrence or multifocal lesions according to mutational and immunohistochemical assessment, and the authors found that the cases with multifocal lesions showed a longer interval between the initial and secondary lesions and a lower cumulative recurrence rate after resection of remnant pancreatic lesions compared with the cases with recurrence.^10^

Although the difference was not statistically significant, the survival time after both the initial pancreatic resection and the diagnosis of remnant pancreatic cancer tended to be longer in the early‐stage group than in the advanced‐stage group. One possible explanation may be that the remnant pancreatic lesions in the advanced‐stage group included more recurrent lesions than those in the early‐stage group. If a remnant pancreatic lesion is due to recurrence, then the prognosis might be poorer than in the case of multifocal lesions.^10^ In contrast, a pooled analysis by Zhou et al in patients who underwent a second pancreatectomy for remnant pancreatic cancer showed the opposite result.^18^ In their study, patients whose initial cancer was UICC stage IA‐IB had a tendency for lower OS than those whose initial cancer was UICC stage IIA or greater, although the difference was not statistically significant (5‐year OS 27.7% vs 45.2%; *P* = .968). Whether the stage of the initial cancer influences the prognosis of the remnant pancreatic cancer is unclear. Further analyses, such as a multicenter study or genetic analyses, are necessary to address this question.

As with primary pancreatic cancer, the treatment options for remnant pancreatic cancer include surgical resection, chemotherapy, and chemoradiotherapy. Our results suggested that surgical resection provides a favorable outcome, if indicated. Suzuki et al analyzed 23 cases of remnant pancreatic cancer after resection of PDAC, and the results showed a significantly better prognosis for patients who underwent surgical resection compared with those who received non‐surgical treatment.^19^ A project study conducted by the Japanese Society of Hepato‐Biliary‐Pancreatic Surgery also showed that resected cases of remnant pancreatic cancer had a significantly longer survival time compared with unresected cases.^20^ Because of adhesion and changes of anatomy, surgery for remnant pancreatic cancer is challenging. However, the project study also showed that morbidity (Clavien–Dindo classification ≥ III) and 30‐day mortality rates after surgical resection of remnant pancreatic cancer were 9% and 0%, respectively. These rates are acceptable considering the outcomes of pancreatic resection according to the nationwide database.^21^, ^22^ Therefore, surgical resection for remnant pancreatic cancer may be feasible, if indicated.

The current study has several limitations. Because pathological findings were collected from pathology reports in this study, the status of pancreatic intraepithelial neoplasia (PanIN) in the background pancreas was not assessed. Matsuda et al reported that PDAC with concomitant IPMN was an independent risk factor for remnant pancreatic cancer and that concomitant PDAC and IPMN showed higher PanIN density in the background pancreas than PDAC without concomitant IPMN.^9^ These findings suggest that PanIN density might be correlated with the incidence of remnant pancreatic cancer. Assessing PanIN density of the background pancreas might be helpful when evaluating the potential of developing remnant pancreatic cancer. In addition, this was a single‐institutional retrospective study based on data from clinical records, and the size of the cohort was not large. Additionally, some patients who developed remnant pancreatic cancer may have been missed because some patients were lost during follow‐up or because diagnostic imaging devices in the early study period were not sophisticated enough to detect small lesions in the remnant pancreas. A multi‐institutional prospective follow‐up study of patients after pancreatectomy for pancreatic cancer may be ideal for determining the nature of remnant pancreatic lesions after surgery for pancreatic cancer.

In conclusion, this was the first study to analyze the incidence of remnant pancreatic cancer according to the degree of progression of the initial cancer. The potential for developing remnant pancreatic cancer was comparable between the early‐stage and the advanced‐stage groups. The lower incidence of remnant pancreatic cancer in the advanced‐stage group may be attributable to shorter observation periods secondary to the poor prognosis of this group. Therefore, we expect that the incidence of remnant pancreatic cancer will increase if long‐term outcomes of patients who undergo surgery for pancreatic cancer are improved owing to progress in multidisciplinary treatment. In the near future, long‐term surveillance after pancreatic resection for pancreatic cancer will be more important to detect remnant pancreatic cancer in a resectable state.

## DISCLOSURE

Funding: This study was supported by Grants‐in‐Aid for Scientific Research (KAKENHI; #19K09175).

Conflict of Interest: The authors declare no conflict of interests related to this article.

Ethical Approval: This study was approved by an institutional review board of Kyushu University.
